# (μ-4-Bromo-2-{1-[2-(dimethyl­amino)ethyl­imino]eth­yl}phenolato)bis­[ethyl­zinc(II)]

**DOI:** 10.1107/S1600536807065208

**Published:** 2007-12-06

**Authors:** Wen-Chou Hung, Shu-Ling Lai, Chu-Chieh Lin

**Affiliations:** a Department of Chemistry, National Chung Hsing University, Taichung 402, Taiwan; b College of Design, Ling Tung University, Taichung 408, Taiwan

## Abstract

The title complex, [Zn_2_(C_2_H_5_)_2_(C_12_H_16_BrN_2_O)_2_], is dimeric, bridged through the O atoms of the phenolate anions. The molecule lies on a crystallographic twofold rotation axis. Each Zn atom is penta­coordinated by two N atoms and two bridging O atoms of the tridentate salicylideneiminate ligands and one C atom from an ethyl group, forming a distorted square-pyramidal environment.

## Related literature

For related literature, see: Chamberlain *et al.* (2001 ▶); Chen *et al.* (2005 ▶, 2006 ▶); Chisholm *et al.* (2000 ▶); Dechy-Cabaret *et al.* (2004 ▶); Gref *et al.* (1994 ▶); Jeong *et al.* (1997 ▶); Williams *et al.* (2003 ▶); Wu *et al.* (2005 ▶, 2006 ▶)
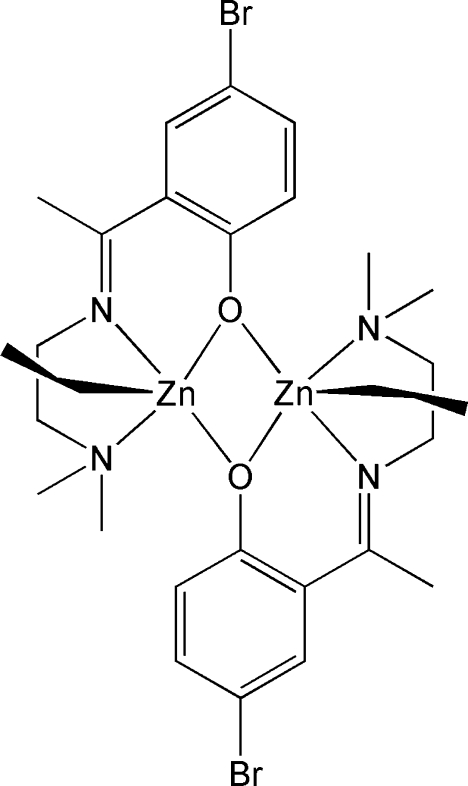


## Experimental

### 

#### Crystal data

[Zn_2_(C_2_H_5_)_2_(C_12_H_16_BrN_2_O)_2_]
*M* *_r_* = 757.22
Orthorhombic, 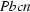

*a* = 21.656 (6) Å
*b* = 7.839 (2) Å
*c* = 19.114 (5) Å
*V* = 3244.9 (14) Å^3^
*Z* = 4
Mo *K*α radiation
μ = 3.97 mm^−1^
*T* = 293 (2) K
0.17 × 0.16 × 0.15 mm

#### Data collection

Bruker SMART 1K CCD diffractometer
Absorption correction: none
17313 measured reflections
3207 independent reflections
1957 reflections with *I* > 2σ(*I*)
*R*_int_ = 0.086

#### Refinement

*R*[*F* ^2^ > 2σ(*F* ^2^)] = 0.040
*wR*(*F* ^2^) = 0.107
*S* = 1.00
3207 reflections
172 parameters
H-atom parameters constrained
Δρ_max_ = 0.68 e Å^−3^
Δρ_min_ = −0.39 e Å^−3^

### 

Data collection: *SMART* (Bruker, 1999 ▶); cell refinement: *SAINT* (Bruker, 1999 ▶); data reduction: *SAINT*; program(s) used to solve structure: *SHELXS97* (Sheldrick, 1997 ▶); program(s) used to refine structure: *SHELXL97* (Sheldrick, 1997 ▶); molecular graphics: *SHELXTL* (Bruker, 1999 ▶); software used to prepare material for publication: *SHELXTL*.

## Supplementary Material

Crystal structure: contains datablocks global, I. DOI: 10.1107/S1600536807065208/at2521sup1.cif

Structure factors: contains datablocks I. DOI: 10.1107/S1600536807065208/at2521Isup2.hkl

Additional supplementary materials:  crystallographic information; 3D view; checkCIF report

## Figures and Tables

**Table:** 

Zn—C13	2.022 (4)
Zn—O1^i^	2.060 (3)
Zn—O1	2.142 (3)
Zn—N1	2.180 (4)
Zn—N2	2.236 (4)

**Table:** 

C13—Zn—O1^i^	119.14 (15)
C13—Zn—O1	113.25 (15)
O1^i^—Zn—O1	74.93 (13)
C13—Zn—N1	110.12 (16)
O1^i^—Zn—N1	129.91 (12)
O1—Zn—N1	78.18 (13)
C13—Zn—N2	114.09 (17)
O1^i^—Zn—N2	89.20 (13)
O1—Zn—N2	131.93 (14)
N1—Zn—N2	78.48 (14)

Symmetry code: (i) 
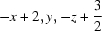
.

**Fig. 1.**
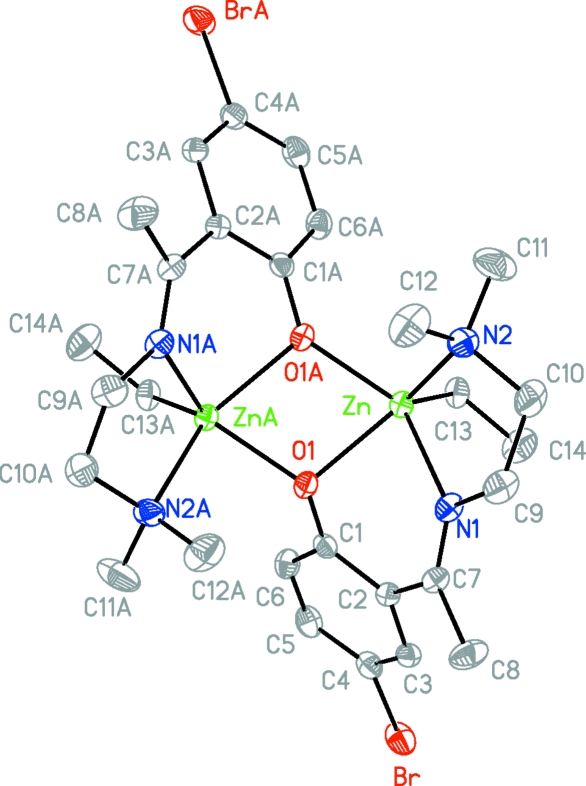
A view of the molecular structure of (I) with displacement ellipsoids shown at the 20% probability level.

## supplementary crystallographic information

### Comment

Poly(ε-caprolactone) (PCL) and poly(lactide) (PLA) and their copolymers have been attracting considerable attention due to their potential applications in many fields (Gref *et al.*,1994; Jeong *et al.*, 1997). The major polymerization method employed to synthesize these polymers is the ring-opening polymerization (ROP) of lactones/lactides. Many zinc complexes with various ligands have been reported to be effective initiators/catalyst for ROP of lactones/lactides (Chamberlain *et al.*, 2001; Williams *et al.*, 2003; Dechy-Cabaret *et al.* 2004; Chen, *et al.*, 2005, Wu, *et al.*, 2005; Wu *et al.*, 2006). Tripodal tridentate ligand supported zinc complexes have been synthesized and used for the polymerization of lactides and the polymerizations are living with relatively low polydispersities (Chisholm *et al.*, 2000). Recently, we have synthesized a series of Schiff base zinc complexes which have shown high activity in the ROP of lactide (Chen *et al.*, 2006). We report herein the synthesis and crystal structure of a NNO-tridentate Schifff base zinc complex (I), a potential catalyst for lactide polymerization.

The solid structure of (I) reveals a dimeric Zn(II) complex (Fig. 1.) containing a Zn_2_O_2_ core bridging through the oxygen atoms of the phenolate. The geometry around Zn atom is pentacoordinated with a distorted square pyramid geometry in which two nitrogen atoms and two oxygen atoms are almost coplanar occupied the basal positions. The ethyl group is sitting on the axial position. The zinc atom is *ca* 0.888 Å above the O1/O1A/N1/N2 mean plane. The distances between the Zn atom and O1, O1A, N1, N2, and C13 are 2.142 (3), 2.060 (3), 2.180 (4), 2.236 (4), 2.022 (4) Å, respectively, which are all within a normal range for Schiff base Zn(II) complexes (Chen *et al.*, 2006).

### Experimental

The ligand, 4-bromo-2-[1-(2-dimethylamino-ethylimino)ethyl]phenol was prepared by the reaction of 2-dimethylaminoethylamine (1.39 g, 22 mmol) with 5-bromo-2-hydroxyacetophenone (4.30 g, 20 mmol) in ethanol (30 ml) at room temperature for 24 h. Volatile materials were removed under vacuum and the resulting material was dissolved in hot hexane (30 ml). The solution was then cooled at 250 K for 24 h giving yellow powder.

The title complex was synthesized by the following procedures. To an ice cold solution (273 K) of 4-bromo-2-[1-(2-dimethylamino-ethylimino)ethyl]phenol (0.57 g, 2.0 mmol) in 40 ml hexane was slowly added a diethyl zinc (2.2 ml, 1 *M* in hexane, 2.2 mmol) solution. The mixture was stirred at room temoerature for 3 h during which the formation of yellow precipitate was observed. The resulting solid was collected by filtration and then dried under vacuum to give yellow powder. Yellow crystals was obtained from the recrystallization of a mixed dichloromethane/hexane solution.

### Refinement

All non-H atoms were initially located in a difference Fourier map. The methyl H atoms were then constrained to an ideal geometry with C—H distances of 0.96 Å and *U*_iso_(H) = 1.5*U*_eq_(C), but each group was allowed to rotate freely about its C—C bond. All other H atoms were placed in geometrically idealized positions and constrained to ride on their parent atoms with C—H distances in the range 0.95–1.00 Å and *U*_iso_(H) =1.2*U*_eq_(C).

### Crystal data

**Table:**

[Zn_2_(C_2_H_5_)_2_(C_12_H_16_BrN_2_O)_2_]	*F*_000_ = 1536
*M**_r_* = 757.22	*D*_x_ = 1.550 Mg m^−^^3^
Orthorhombic, *P**b**c**n*	Mo *K*α radiation λ = 0.71073 Å
Hall symbol: -P 2n 2ab	Cell parameters from 3732 reflections
*a* = 21.656 (6) Å	θ = 2.8–24.0º
*b* = 7.839 (2) Å	µ = 3.97 mm^−^^1^
*c* = 19.114 (5) Å	*T* = 293 (2) K
*V* = 3244.9 (14) Å^3^	Parallelpiped, yellow
*Z* = 4	0.17 × 0.16 × 0.15 mm

### Data collection

**Table:**

Bruker SMART 1K CCD diffractometer	1957 reflections with *I* > 2σ(*I*)
Radiation source: fine-focus sealed tube	*R*_int_ = 0.086
Monochromator: graphite	θ_max_ = 26.1º
*T* = 298(2) K	θ_min_ = 1.9º
φ and ω scans	*h* = −26→25
Absorption correction: none	*k* = −9→9
17313 measured reflections	*l* = −13→23
3207 independent reflections	

### Refinement

**Table:**

Refinement on *F*^2^	Secondary atom site location: difference Fourier map
Least-squares matrix: full	Hydrogen site location: inferred from neighbouring sites
*R*[*F*^2^ > 2σ(*F*^2^)] = 0.040	H-atom parameters constrained
*wR*(*F*^2^) = 0.107	*w* = 1/[σ^2^(*F*_o_^2^) + (0.052*P*)^2^] where *P* = (*F*_o_^2^ + 2*F*_c_^2^)/3
*S* = 1.00	(Δ/σ)_max_ = 0.002
3207 reflections	Δρ_max_ = 0.68 e Å^−^^3^
172 parameters	Δρ_min_ = −0.39 e Å^−^^3^
Primary atom site location: structure-invariant direct methods	Extinction correction: none

### Special details

**Table:**

Geometry. All e.s.d.'s (except the e.s.d. in the dihedral angle between two l.s. planes) are estimated using the full covariance matrix. The cell e.s.d.'s are taken into account individually in the estimation of e.s.d.'s in distances, angles and torsion angles; correlations between e.s.d.'s in cell parameters are only used when they are defined by crystal symmetry. An approximate (isotropic) treatment of cell e.s.d.'s is used for estimating e.s.d.'s involving l.s. planes.
Refinement. Refinement of *F*^2^ against ALL reflections. The weighted *R*-factor *wR* and goodness of fit *S* are based on *F*^2^, conventional *R*-factors *R* are based on *F*, with *F* set to zero for negative *F*^2^. The threshold expression of *F*^2^ > σ(*F*^2^) is used only for calculating *R*-factors(gt) *etc*. and is not relevant to the choice of reflections for refinement. *R*-factors based on *F*^2^ are statistically about twice as large as those based on *F*, and *R*- factors based on ALL data will be even larger.

### Fractional atomic coordinates and isotropic or equivalent isotropic displacement parameters (Å^2^)

**Table:**

	*x*	*y*	*z*	*U*_iso_*/*U*_eq_	
Zn	0.93264 (2)	0.18811 (6)	0.70779 (3)	0.04040 (17)	
Br	0.79595 (3)	0.47079 (8)	1.01986 (3)	0.0781 (2)	
O1	0.97357 (12)	0.1816 (4)	0.80980 (15)	0.0476 (8)	
N1	0.87340 (16)	0.0062 (4)	0.7620 (2)	0.0462 (9)	
N2	0.92031 (17)	−0.0132 (5)	0.6261 (2)	0.0569 (10)	
C1	0.93610 (19)	0.2386 (6)	0.8581 (2)	0.0424 (10)	
C2	0.87745 (18)	0.1621 (5)	0.8681 (2)	0.0395 (10)	
C3	0.8368 (2)	0.2322 (6)	0.9182 (2)	0.0461 (11)	
H3A	0.7984	0.1821	0.9254	0.055*	
C4	0.8534 (2)	0.3742 (6)	0.9567 (2)	0.0536 (12)	
C5	0.9115 (2)	0.4456 (6)	0.9490 (3)	0.0613 (14)	
H5A	0.9231	0.5387	0.9761	0.074*	
C6	0.9519 (2)	0.3770 (6)	0.9005 (3)	0.0566 (13)	
H6A	0.9911	0.4247	0.8961	0.068*	
C7	0.85761 (18)	0.0141 (5)	0.8261 (3)	0.0428 (10)	
C8	0.8185 (3)	−0.1206 (7)	0.8618 (3)	0.0779 (17)	
H8A	0.8087	−0.2092	0.8289	0.117*	
H8B	0.7810	−0.0697	0.8786	0.117*	
H8C	0.8410	−0.1683	0.9003	0.117*	
C9	0.8539 (2)	−0.1357 (6)	0.7177 (3)	0.0655 (15)	
H9A	0.8114	−0.1653	0.7279	0.079*	
H9B	0.8795	−0.2348	0.7269	0.079*	
C10	0.8600 (2)	−0.0839 (7)	0.6419 (3)	0.0780 (18)	
H10A	0.8529	−0.1827	0.6125	0.094*	
H10B	0.8286	0.0002	0.6310	0.094*	
C11	0.9207 (3)	0.0648 (9)	0.5561 (3)	0.104 (2)	
H11A	0.9154	−0.0223	0.5213	0.156*	
H11B	0.9593	0.1219	0.5486	0.156*	
H11C	0.8875	0.1457	0.5526	0.156*	
C12	0.9678 (3)	−0.1460 (7)	0.6266 (4)	0.092 (2)	
H12A	0.9593	−0.2274	0.5903	0.138*	
H12B	0.9677	−0.2026	0.6711	0.138*	
H12C	1.0075	−0.0952	0.6186	0.138*	
C13	0.88570 (19)	0.4061 (5)	0.6884 (2)	0.0469 (11)	
H13A	0.9059	0.4986	0.7132	0.056*	
H13B	0.8885	0.4307	0.6388	0.056*	
C14	0.8200 (2)	0.4042 (8)	0.7084 (3)	0.0851 (19)	
H14A	0.8016	0.5121	0.6970	0.128*	
H14B	0.8165	0.3843	0.7578	0.128*	
H14C	0.7992	0.3150	0.6834	0.128*	

### Atomic displacement parameters (Å^2^)

**Table:**

	*U*^11^	*U*^22^	*U*^33^	*U*^12^	*U*^13^	*U*^23^
Zn	0.0380 (3)	0.0341 (3)	0.0490 (3)	0.0014 (2)	−0.0007 (2)	−0.0017 (2)
Br	0.0802 (4)	0.0978 (5)	0.0562 (4)	0.0351 (3)	−0.0039 (3)	−0.0238 (3)
O1	0.0353 (15)	0.0560 (19)	0.052 (2)	0.0020 (14)	0.0015 (13)	−0.0005 (15)
N1	0.047 (2)	0.032 (2)	0.061 (3)	−0.0057 (16)	0.0050 (18)	−0.0076 (18)
N2	0.060 (3)	0.055 (2)	0.055 (3)	−0.012 (2)	0.0055 (19)	−0.019 (2)
C1	0.046 (2)	0.044 (2)	0.037 (3)	0.006 (2)	−0.005 (2)	0.002 (2)
C2	0.043 (2)	0.037 (2)	0.039 (2)	0.0009 (19)	0.0002 (19)	0.0008 (19)
C3	0.045 (2)	0.051 (3)	0.042 (3)	0.003 (2)	0.001 (2)	0.002 (2)
C4	0.056 (3)	0.062 (3)	0.043 (3)	0.020 (2)	−0.003 (2)	−0.005 (2)
C5	0.069 (3)	0.051 (3)	0.064 (4)	0.001 (3)	−0.016 (3)	−0.012 (3)
C6	0.050 (3)	0.056 (3)	0.064 (4)	−0.006 (2)	−0.006 (2)	−0.007 (3)
C7	0.041 (2)	0.035 (2)	0.052 (3)	−0.0002 (19)	0.007 (2)	0.001 (2)
C8	0.083 (4)	0.068 (4)	0.083 (4)	−0.030 (3)	0.024 (3)	0.003 (3)
C9	0.072 (3)	0.046 (3)	0.078 (4)	−0.022 (3)	0.015 (3)	−0.025 (3)
C10	0.065 (4)	0.079 (4)	0.089 (5)	−0.029 (3)	0.006 (3)	−0.041 (3)
C11	0.143 (6)	0.114 (5)	0.054 (4)	−0.046 (5)	0.003 (4)	−0.021 (4)
C12	0.083 (4)	0.065 (4)	0.128 (6)	0.003 (3)	0.020 (4)	−0.032 (4)
C13	0.046 (2)	0.029 (2)	0.066 (3)	0.0080 (19)	−0.010 (2)	0.003 (2)
C14	0.073 (4)	0.063 (4)	0.119 (5)	0.021 (3)	0.015 (4)	0.027 (4)

### Geometric parameters (Å, °)

**Table:**

Zn—C13	2.022 (4)	C6—H6A	0.9300
Zn—O1^i^	2.060 (3)	C7—C8	1.516 (6)
Zn—O1	2.142 (3)	C8—H8A	0.9600
Zn—N1	2.180 (4)	C8—H8B	0.9600
Zn—N2	2.236 (4)	C8—H8C	0.9600
Br—C4	1.892 (4)	C9—C10	1.509 (7)
O1—C1	1.307 (5)	C9—H9A	0.9700
O1—Zn^i^	2.060 (3)	C9—H9B	0.9700
N1—C7	1.273 (5)	C10—H10A	0.9700
N1—C9	1.460 (6)	C10—H10B	0.9700
N2—C10	1.451 (6)	C11—H11A	0.9600
N2—C12	1.464 (6)	C11—H11B	0.9600
N2—C11	1.471 (7)	C11—H11C	0.9600
C1—C6	1.398 (6)	C12—H12A	0.9600
C1—C2	1.417 (6)	C12—H12B	0.9600
C2—C3	1.413 (6)	C12—H12C	0.9600
C2—C7	1.475 (6)	C13—C14	1.472 (7)
C3—C4	1.382 (6)	C13—H13A	0.9700
C3—H3A	0.9300	C13—H13B	0.9700
C4—C5	1.384 (6)	C14—H14A	0.9600
C5—C6	1.383 (7)	C14—H14B	0.9600
C5—H5A	0.9300	C14—H14C	0.9600
			
C13—Zn—O1^i^	119.14 (15)	C7—C8—H8A	109.5
C13—Zn—O1	113.25 (15)	C7—C8—H8B	109.5
O1^i^—Zn—O1	74.93 (13)	H8A—C8—H8B	109.5
C13—Zn—N1	110.12 (16)	C7—C8—H8C	109.5
O1^i^—Zn—N1	129.91 (12)	H8A—C8—H8C	109.5
O1—Zn—N1	78.18 (13)	H8B—C8—H8C	109.5
C13—Zn—N2	114.09 (17)	N1—C9—C10	109.1 (4)
O1^i^—Zn—N2	89.20 (13)	N1—C9—H9A	109.9
O1—Zn—N2	131.93 (14)	C10—C9—H9A	109.9
N1—Zn—N2	78.48 (14)	N1—C9—H9B	109.9
C1—O1—Zn^i^	136.0 (3)	C10—C9—H9B	109.9
C1—O1—Zn	112.2 (2)	H9A—C9—H9B	108.3
Zn^i^—O1—Zn	105.00 (13)	N2—C10—C9	112.4 (4)
C7—N1—C9	121.2 (4)	N2—C10—H10A	109.1
C7—N1—Zn	125.7 (3)	C9—C10—H10A	109.1
C9—N1—Zn	113.1 (3)	N2—C10—H10B	109.1
C10—N2—C12	111.1 (4)	C9—C10—H10B	109.1
C10—N2—C11	110.7 (5)	H10A—C10—H10B	107.8
C12—N2—C11	107.3 (5)	N2—C11—H11A	109.5
C10—N2—Zn	103.4 (3)	N2—C11—H11B	109.5
C12—N2—Zn	114.4 (3)	H11A—C11—H11B	109.5
C11—N2—Zn	110.0 (3)	N2—C11—H11C	109.5
O1—C1—C6	121.5 (4)	H11A—C11—H11C	109.5
O1—C1—C2	120.4 (4)	H11B—C11—H11C	109.5
C6—C1—C2	118.0 (4)	N2—C12—H12A	109.5
C3—C2—C1	119.1 (4)	N2—C12—H12B	109.5
C3—C2—C7	119.6 (4)	H12A—C12—H12B	109.5
C1—C2—C7	121.4 (4)	N2—C12—H12C	109.5
C4—C3—C2	120.8 (4)	H12A—C12—H12C	109.5
C4—C3—H3A	119.6	H12B—C12—H12C	109.5
C2—C3—H3A	119.6	C14—C13—Zn	115.4 (3)
C3—C4—C5	120.4 (4)	C14—C13—H13A	108.4
C3—C4—Br	119.4 (4)	Zn—C13—H13A	108.4
C5—C4—Br	120.2 (4)	C14—C13—H13B	108.4
C4—C5—C6	119.3 (5)	Zn—C13—H13B	108.4
C4—C5—H5A	120.3	H13A—C13—H13B	107.5
C6—C5—H5A	120.3	C13—C14—H14A	109.5
C1—C6—C5	122.3 (4)	C13—C14—H14B	109.5
C1—C6—H6A	118.8	H14A—C14—H14B	109.5
C5—C6—H6A	118.8	C13—C14—H14C	109.5
N1—C7—C2	118.9 (4)	H14A—C14—H14C	109.5
N1—C7—C8	123.3 (4)	H14B—C14—H14C	109.5
C2—C7—C8	117.8 (4)		

Symmetry codes: (i) −*x*+2, *y*, −*z*+3/2.
